# A survey on clinical pathways of patients with epilepsy and cerebrovascular diseases or brain tumors

**DOI:** 10.1007/s10072-020-04252-5

**Published:** 2020-01-18

**Authors:** Gaetano Zaccara, Vincenzo Esposito, Marta Maschio, Rosa Musolino, Roberta Rudà, Danilo Toni

**Affiliations:** 1 Regional Health Agency of Tuscany, via Pietro Dazzi 1, 50141 Florence, Italy; 2 Neurosurgery, IRCCS Neuromed, Pozzilli, IS Italy; 3 Department of Human Neurosciences, Sapienza University, Rome, Italy; 4 Center for Brain Tumor-Related Epilepsy, UOSD Neurology, I.R.C.C.S. IFO- Regina Elena National Cancer Institute, Rome, Italy; 5 U.O.S.D. Stroke Unit, Department of Clinical and Experimental Medicine, AOU Policlinico G: Martino, University of Messina, Messina, Italy; 6 Department of Neuro-Oncology, University and City of Health and Science, Turin, Italy; 7 Department of Human Neurosciences, Sapienza University, Rome, Italy

**Keywords:** Epilepsy, Seizures, Clinical pathways, Cerebrovascular epilepsy, Brain tumor epilepsy, Survey, Multidisciplinary approach

## Abstract

**Objective:**

Patients with seizures and epilepsies comorbid with cerebrovascular disorders (CVDs) or brain tumors (BTs) are managed by different specialists, including neurologists with expertise in epilepsy (epileptologists), CVDs, and neuro-oncology, as well as neurologists without special expertise (general neurologists), and also emergency room physicians (EPs), intensive care physicians, and neurosurgeons. It has never been studied how these specialists interact for the treatment of seizures or epilepsy in these patients.

**Methods:**

A survey was used to investigate how patients with such comorbidities are managed in hospitals in Italy.

**Results:**

One hundred and twenty-eight specialists from hospitals in all parts of Italy filled in a questionnaire. Epileptologists were in charge of treatment of epilepsy in about 50% of cases while acute seizures were treated mainly by general neurologists (52% of cases). Diagnostic, therapeutic, and assistance pathways (PDTAs) for CVD and BT epilepsies were declared by physicians in about half of the hospitals while in about a quarter, there were only informal agreements and, in the remaining hospitals, there were no agreements between specialists. CVD neurologists, specialists in internal medicine, and EP were most often in charge of treatment of epilepsy comorbid with CVD. General neurologists, neuro-oncologists, and neurosurgeons were included in teams that manage BT epilepsies while epileptologists were included only in a small percentage of hospitals.

**Conclusions:**

Clinical decisions on epilepsy or seizures in patients with such comorbidities are often handled by different specialists. A new team culture and PDTAs are needed to guarantee high standards of diagnostic and therapeutic procedures.

## Introduction

In recent years, the prevalence of patients with epilepsy comorbid with other chronic diseases (a greater than coincidental association of two conditions in the same individual) (1) has consistently increased (2, 3). Within this context, comorbidity between acute seizures or epilepsy and cerebrovascular diseases (CVDs) or brain tumors (BTs) is of particular relevance.

Incidence of acute symptomatic seizures in stroke patients lies between 3 and 6% (4) while the incidence is up to 10–16% of cases (5) in patients with intracranial (intracerebral or subarachnoid) hemorrhages. In addition, long-term follow-up studies suggest incidence of post stroke epilepsy (remote unprovoked seizures) to be between 10 and 12% (6). Several data indicate a complex relationship between these two diseases (7).

Incidence of epilepsy in patients with BTs varies from 35 to 70% (8–10) with seizures being the most common symptom in such patients, while a BT is associated with epilepsy in 6–10% of all patients with epilepsy (11).

These comorbid diseases are managed by different specialists, including neurologists without special expertise in these specific fields (general neurologists), neurologists with special expertise in CVDs, in epilepsy (epileptologists), neuro-oncologists, emergency room physicians (EPs), intensive care physicians, neurosurgeons, oncologists, radiotherapists, etc. Although these conditions require a multidisciplinary approach, there is often a lack of effective coordination of all interventions.

Recently, a survey investigated how patients with epileptic seizures or status epilepticus are managed in emergency and in the subsequent hospital pathways in Italy (12). It emerged that epileptologists were in charge of a small percentage of such cases and there was a high variability in the specialists involved in hospital treatment of epilepsy.

Here, the results of a second survey investigating how patients with epilepsy or seizures and the above reported comorbidities are managed in Italy are reported. Administrative data cannot give us this information because there is no way to distinguish between consultancy provided by general neurologists, epileptologists, and neurologists with expertise in CVDs.

## Material and methods

On September 2018, a meeting was organized to discuss the clinical pathways of patients with comorbidities between CVDs or BTs and epileptic seizures. This meeting took place in six Italian cities (Mestre, Messina, Milano, Napoli, Roma, Siena) which were connected to each other via web conference. Neurologists (general neurologists, epileptologists, neuro-oncologists, CVD neurologists), neurosurgeons, EP, and intensivists who were actively involved in the hospital treatment of these patients were invited from all parts of Italy.

Before the meeting, all invited participants had to fill in a questionnaire through an electronic system that guaranteed anonymity. The questionnaire (see supplementary material, Table S1) comprised of three groups of questions which were structured to collect information on how patients with BTs or CVDs and seizures were managed in their hospitals. In the first group of questions (Qs = 1, 2), participants were asked to state the professional involved in the treatment of seizures or epilepsies in patients with such comorbidities both in acute and chronic settings. In the second and third group of questions, participants were asked to indicate whether formal diagnostic, therapeutic and assistance pathways (PDTAs), or only informal agreements were available for patients with seizures and CVDs (Qs = 3, 4, 5, 6) or BTs (Qs = 7, 8, 9, 10) and which professionals were involved in their treatment. A *PDTA* means an official document approved by hospital teams and health management of each hospital. An *informal agreement* means that there was no written document but only verbal agreements between hospital teams involved in the management of these patients. The last two questions were open-ended and participants were required to indicate what they thought was the most important critical issue in the clinical pathway of such patients (Q = 11) and to make a proposal for improving the indicated critical issue (Q = 12). For each question, participants could skip a specific question if they had no opinion on the issue.

Because this was a descriptive survey, all variables were analyzed using only descriptive statistics.

## Results

A total of 128 invited participants (75 females; mean age 49 years) answered the questionnaire (110 neurologists, 11 neurosurgeons, 5 intensivists, 2 other specialists).

**Fig. 1:**
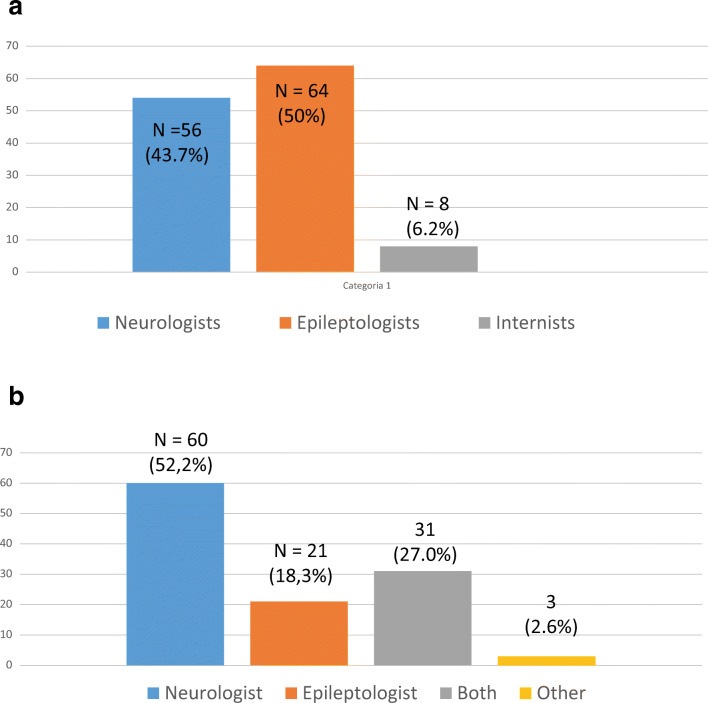
**a** In your hospital which physician is in charge of chronic treatment of patients with epilepsy? (n = 128) **b** In your hospital which physician takes treatment decisions for patients with seizures? (n = 115)*

Answers to questions concerning which specialist is in charge of chronic treatment of people with epilepsy or takes treatment decisions for patients with seizures are reported in Fig. 1a and 1b.

### PDTA of the multidisciplinary team that take care of patients with CVDs and seizures

Sixty-five participants (50.8%) answered that in their hospital, there was a PDTA for patients with CVDs. Within the group without a formal pathway (*n* = 63), 24 participants (38.1%) affirmed that there were informal agreements, and 39 participants (61.9%) declared a lack of any kind of agreement between clinicians involved in the treatment of patients with such comorbidity. Table 1 reports those specialists included in formal clinical pathways or in informal agreements.

**Table 1:** Specialists included in a PDTA for patients with epilepsy and CVD, according to the opinions of participants

	Specialists included in a PDTA (*n* = 65)*	Specialists involved in informal agreements (*n* = 24)*
General neurologists and epileptologists	18 (27.7%)	6 (25%)
Epileptologists, CVD neurologists, specialists in internal medicine, EPs	22 (33.8%)	11 (45.8%)
General neurologists, CVD neurologists, specialists in internal medicine, EPs	21 (32.3%)	3 (12.5%)
General neurologists, emergency physicians	3 (4.6%)	4 (16.7%)
CVD neurologists, specialists in internal medicine, EPs	1 (1.5%)	0
Other specialists	0	0

*Total number of participants who answered each specific question

### PDTA of the multidisciplinary team that take care of patients with BTs and seizures

Fifty-eight participants (45.3%) answered that in their hospital, there was a formal PDTA. Within the group of those without a formal pathway (*n* = 70), 39 participants (55.7%) had informal agreements, and 31 participants (44.3%) affirmed that in their hospital, there was a lack of any agreement between hospital teams involved in the management of these patients. Table 2 reports those specialists included in formal clinical pathways or in informal agreements.

**Table 2:** Specialists included in a PDTA for patients with BT and seizures, according to the opinions of participants

	Specialists included in a PDTA (*n* = 58)*	Specialists involved in informal agreements (*n* = 39)*
General neurologists, epileptologists, and neurosurgeons	8 (13.8)	10 (25.6%)
General neurologists, neuro-oncologists, neurosurgeons	15 (25.9%)	18 (46.1%)
General neurologists, neuro-oncologists, neurosurgeons, EPs	24 (41.4%)	4 (10.2%)
Neurosurgeons, neuro-oncologists, EPs	6 (10.3%)	2 (5.1%)
General neurologists, neuro-oncologists, EPs	1 (1.7%)	3 (7.7%)
Other specialists	4 (6.8%)	2 (5.1%)

*Total number of participants who answered each specific question

The two open questions concerning what the participants thought was the most important critical issue for the treatment of such patients and their proposals to improve the above reported critical issue had 115 and 110 answers, respectively.

Forty-six participants (40%) answered that the most important criticalities concerned organizational issues, 30 (26%) focused on staff or facility shortages (in 10 cases unavailability of EEG in the emergency departments), and 11 (9.6%) focused on poor knowledge of epilepsy among physicians involved in the multidisciplinary team. The remaining 28 participants (24.3%) answered that there were no criticalities or gave different answers. Of 110 participants who made proposals for improvement, 40 (36.3%) focused on constitution or improvement of clinical pathways, 25 (22.7%) suggested more facilities (in 6 cases availability of EEG in the emergency), and 11 (10%) more training between physicians. No specific proposal or different proposals were given by 34 (30.9%) participants. From the inspection of answers, there were some differences between participants. Participants from the north of Italy focused more on organizational issues and those from the south of Italy focused on lack of facilities.

## Discussion

This survey was filled by neurologists with different expertise (general neurologists, epileptologists, neuro-oncologists, CVD neurologists) and by other specialists. All these specialists were working in different hospitals in Italy; hence, this survey gives a relatively precise picture of how patients with epilepsy and such comorbidities are managed in Italy.

The main results can be summarized in the following order:

1. While epileptologists are most often involved in the treatment of patients with epilepsy in the chronic setting, treatment of seizures in the acute setting is most often performed by general neurologists (see Fig. 1). These data confirm our previous finding that showed that treatment of seizures, repetitive seizures, and status epilepticus are managed mainly by general neurologists and not by epileptologists (13).
2. About half of the hospitals had a PDTA both for patients with epilepsy and CVDs and for patients with epilepsy and BTs. For the rest of the hospitals, there were only informal agreements or a lack of any agreement. Again, these findings are very similar to those reported in our previous survey on patients with seizures or status epilepticus (13).
3. There is high variability in professionals included in these pathways.
4. Regarding comorbidity between epilepsy/seizures and CVDs, specialists most often included in the PDTA of such patients were epileptologists or general neurologists, CVD neurologists, specialists in internal medicine, and EPs. Epileptologists were included in the PDTA in about 60% of hospitals while in the remaining 40%, general neurologists and CVD neurologists were included. Other specialists, such as specialists in internal medicine and EPs, were thought to be involved in this pathway in two-third of hospitals. We speculate that in these cases, inclusion of non-neurologists in the management of these patients may be due to the presence of other concomitant comorbidities (for example, cardiac disorders) of patients with CVDs, or the lack of neurologic wards in small hospitals.
5. In the case of patients with epilepsy and BTs, in those hospitals where a PDTA was available, specialists most often included in the pathway were general neurologists, neuro-oncologists, and neurosurgeons while epileptologists were included only in a small percentage of hospitals. In those hospitals where there were only informal agreements, similar specialists were involved with a lower percentage of hospitals including neuro-oncologists. These differences can be explained by the size of each hospital, with the biggest hospitals having formal pathways and availability of neuro-oncologists.

As in our previous survey (13), we speculate that the reason for the observed high variability in specialists involved in these clinical pathways may in part reflect contingent local situations.

In conclusion, it seems that epileptologists in about 60% of hospitals are involved in the treatment of epilepsy or seizures in patients with CVDs, while they are involved in the treatment of BT epilepsy in a much lower percentage of hospitals. In such cases, general neurologists and neuro-oncologists take treatment decisions on seizures in the majority of patients. It is of utmost importance that these specialists should have expertise in the pharmacological treatment of both epilepsy and in the management of the underlying disease.

We conclude that, to achieve high standards of diagnostic and therapeutic procedures, a PDTA for patients with BT and for patients with CVD should be produced with the agreement of all clinicians involved in the treatment of such comorbidities and that these PDTAs should be implemented in all hospitals in which such patients are treated.
